# Livestock and climate change: impact of livestock on climate and mitigation strategies

**DOI:** 10.1093/af/vfy034

**Published:** 2018-11-12

**Authors:** Giampiero Grossi, Pietro Goglio, Andrea Vitali, Adrian G Williams

**Affiliations:** 1 Dipartimento di Scienze Agrarie e Forestali, Università della Tuscia, Viterbo, Italy; 2 School of Water, Energy and Environment, Cranfield University, Cranfield, UK; 3 Facoltà di bioscienze e tecnologie agro-alimentari e ambientali, University of Teramo, Italy

**Keywords:** climate change, greenhouse gases, livestock, mitigation

**Implications**

The livestock sector requires a significant amount of natural resources and has an important role in global greenhouse gas emissions. The most important greenhouse gases from animal agriculture are methane and nitrous oxide.
Mitigation strategies aimed at reducing the emission intensity of this sector are needed to meet the increasing demand for livestock products driven by population growth.
To increase the effectiveness of mitigation strategies, the complex interactions among the components of livestock production systems must be taken into account to avoid environmental trade-offs.

## Introduction

According to the United Nations (UN, 2017), the world population increased by approximately 1 billion inhabitants during the last 12 years, reaching nearly 7.6 billion in 2017. Although this growth is slower than 10 years ago (1.24% vs. 1.10% per year), with an average increase of 83 million people annually, global population will reach about 8.6 billion in 2030 and 9.8 billion in 2050. Population growth, urbanization, and income rise in developing countries are the main driver of the increased demand for livestock products (UN, 2017). The livestock sector requires a significant amount of natural resources and is responsible for about 14.5% of total anthropogenic greenhouse gas emissions (7.1 Gigatonnes of carbon dioxide equivalents for the year 2005; Gerber et al., 2013). Mitigation strategies aimed at reducing emissions of this sector are needed to limit the environmental burden from food production while ensuring a sufficient supply of food for a growing world population. The objectives of this manuscript are to 1) discuss the main greenhouse gas emissions sources from the livestock sector and 2) summarize the best mitigation strategies.

## Impact of Livestock on Climate Change

The most important greenhouse gases from animal agriculture are methane and nitrous oxide. Methane, mainly produced by enteric fermentation and manure storage, is a gas which has an effect on global warming 28 times higher than carbon dioxide. Nitrous oxide, arising from manure storage and the use of organic/inorganic fertilizers, is a molecule with a global warming potential 265 times higher than carbon dioxide. The carbon dioxide equivalent is a standard unit used to account for the global warming potential (IPCC, 2013).

**Figure 1.**
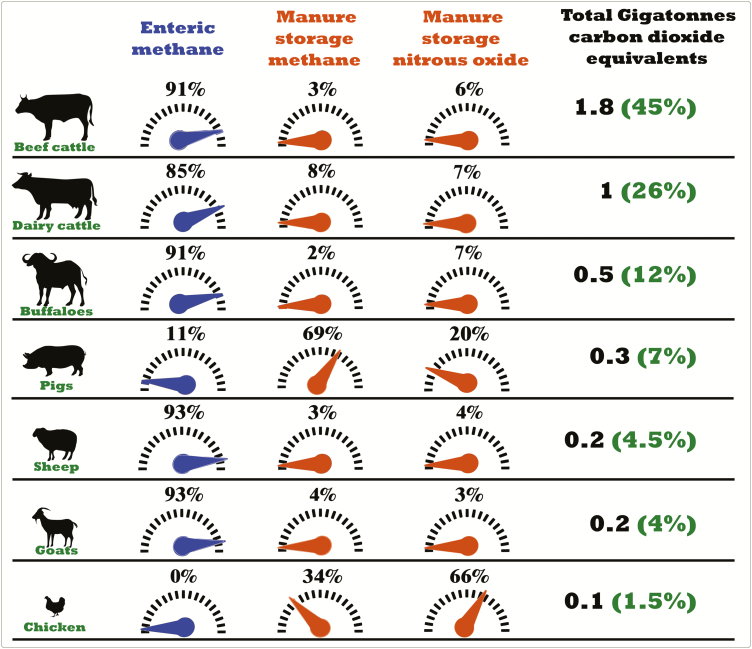
Greenhouse gases incidence of enteric fermentation and manure storage by animal type, expressed as Gigatonnes of carbon dioxide equivalents. Data referred to 2010 (FAO, 2017).

Figure 1 was adapted from the Global Livestock Environmental Assessment Model (GLEAM) developed by FAO (FAO, 2017) and shows in carbon dioxide equivalents the greenhouse gas incidences that enteric fermentation and manure storage have across the main livestock species raised worldwide.

In addition to greenhouse gases arising from enteric fermentation and manure storage, feed production together with the related soil carbon dioxide and nitrous oxide emissions is another important hot spot for the livestock sector. Soil carbon dioxide emissions are due to soil carbon dynamics (e.g., decomposing plant residues, mineralization of soil organic matter, land use change, etc.), the manufacturing of synthetic fertilizers and pesticides, and from fossil fuel use in on-farm agricultural operations (Goglio et al., 2018). Nitrous oxide emissions are emitted when organic and inorganic fertilizers are applied to the soil.

**Figure 2.**
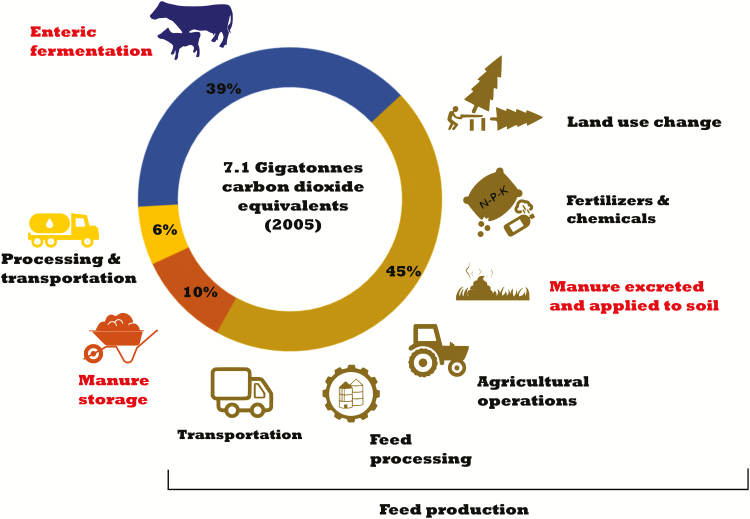
Livestock emissions by source (adapted from Gerber et al., 2013). Direct livestock emissions are shown in red.

As shown in Figure 2, feed production and processing contribute about 45% of the whole sector (3.2 Gigatonnes of carbon dioxide equivalents). Enteric fermentation producing about 2.8 Gigatonnes (39%) is the second largest source of emissions. Manure storage with 0.71 Gigatonnes accounts for about 10% of the total. The remaining 6% (0.42 Gigatonnes of carbon dioxide equivalents) is attributable to the processing and transportation of animal products (Gerber et al., 2013).

Feed production (Figure 2) includes all the greenhouse gas emission arising from 1) land use change, 2) manufacturing and use of fertilizers and pesticides, 3) manure excreted and applied to fields, 4) agricultural operations, 5) feed processing, and 6) feed transport. Although these processes result in a large share of the livestock supply chain, in this article, we mainly focus on direct livestock emissions enteric fermentation, manure storage, and manure excreted/applied to the soil. All other emissions are outside the scope of this article.

### Enteric fermentation

Enteric fermentation is a natural part of the digestive process of ruminants where bacteria, protozoa, and fungi contained in the fore-stomach of the animal (rumen), ferment and break down the plant biomass eaten by the animal. Plant biomass in the rumen is converted into volatile fatty acids, which pass the rumen wall and go to the liver through the circulatory system. This process supplies a major part of the energy needs of the animal and enables the high conversion efficiency of cellulose and semi-cellulose, which is typical of ruminants. The gaseous waste products of enteric fermentation, carbon dioxide and methane, are mainly removed from the rumen by eructation. Methane emission in the reticulorumen is an evolutionary adaptation that enables the rumen ecosystem to dispose hydrogen, which may otherwise accumulate and inhibit carbohydrate fermentation and fiber degradation (McAllister and Newbold, 2008). The emission rate of enteric methane varies according to feed intake and digestibility.

### Manure storage

Manure acts as an emission source for both methane and nitrous oxide, and the quantity emitted is linked to environmental conditions, type of management and composition of the manure. Organic matter and nitrogen content of excreta are the main characteristics influencing emission of methane and nitrous oxide, respectively. Under anaerobic conditions, the organic matter is partially decomposed by bacteria producing methane and carbon dioxide. Storage or treatment of liquid manure (slurry) in a lagoon or tank promotes an anaerobic environment which leads to an increase in methane production. Long storage periods and warm and wet conditions can further increase these emissions (EPA, 2010). On the other hand, nitrous oxide emissions need a combination of aerobic and anaerobic conditions to be produced. Therefore, when manure is handled as a solid (dung) or deposited on pastures, nitrous oxide production increases while little or no methane is emitted. Nitrous oxide is generated through both the nitrification and denitrification processes of the nitrogen contained in manure, which is mainly present in organic form (e.g., proteins) and in inorganic form as ammonium and ammonia. Nitrification occurs aerobically and converts ammonium and ammonia to nitrites and then nitrates, while denitrification occurs anaerobically converting nitrates to nitrous oxide and nitrogen gas (Saggar, 2010). The balance between ammonium and ammonia is highly affected by pH, with ammonia increasing as pH increases.

### Feed production

Almost 60% of the global biomass harvested worldwide enters the livestock subsystem as feed or bedding material (Krausmann et al., 2008). Greenhouse gas emissions from feed production represent 60–80% of the emission coming from eggs, chicken and pork, and 35–45% of the milk and beef sector (Sonesson et al., 2009). As shown in Figure 2, emissions from feed production account for about 45% of the livestock sector. The application of manure as fertilizer for feed crops and the deposition of manure on pastures generates a substantial amount of nitrous oxide emissions representing about half of these emissions (Gerber et al., 2013). Although livestock feed production often involves large applications of nitrogen to agricultural soils, good manure management can reduce the need for manufactured fertilizers.

## Livestock Mitigation Strategies

The extreme heterogeneity of the agricultural sector needs to be taken into account when defining the overall sustainability of a mitigation strategy, which can vary across different livestock systems, species, and climates. Generally, no measure in isolation will encompass the full emission reduction potential, while a combination selected from the full range of existing options will be required to reach the best result (Llonch et al., 2017). It is also important to consider the “pollution swapping” effect when evaluating the effectiveness of a mitigation strategy (Hristov et al., 2013). Reduction of methane emissions during enteric fermentation might be counteracted by increased greenhouse gas emissions in applied manure. Reduction of direct nitrous oxide emissions during storage might result in higher nitrate leaching and ammonia volatilization during field application.

Mitigation may occur directly by reducing the amount of greenhouse gases emitted, or indirectly through the improvement of production efficiency. The main strategies to mitigate greenhouse gas emissions in the livestock sector have been investigated and are summarized in Table 1.

**Table 1.** Mitigation potential of various strategies

Strategies	Category	Potential mitigating effect*	
		Methane	Nitrous Oxide
Enteric fermentation	Forage quality	Low to medium	^†^
	Feed processing	Low	Low
	Concentrate inclusion	Low to medium	^†^
	Dietary lipids	Medium	^†^
	Electrons receptors	High	^†^
	Ionophores	Low	^†^
	Methanogenic inhibitors	Low	^†^
Manure storage	Solid-liquid separation	High	Low
	Anaerobic digestion	High	High
	Decreased storage time	High	High
	Frequent manure removal	High	High
	Phase feeding	^‡^	Low
	Reduced dietary protein	^‡^	Medium
	Nitrification inhibitors	^‡^	Medium to high
	No grazing on wet soil	Low	Medium
	Increased productivity	High	High
Animal management	Genetic selection	High	^‡^
	Animal health	Low to medium	Low to medium
	Increase reproductive eff.	Low to medium	Low to medium
	Reduced animal mortality	Low to medium	Low to medium
	Housing systems	Medium to high	Medium to high

*High = ≥30% mitigating effect; Medium = 10–30% mitigating effect; Low = ≤10% mitigating effect. Mitigating effects refer to percent change over a “standard practice” according to Newell Price et al. (2011); Borhan et al. (2012); Hristov et al. (2013); Montes et al. (2013); Petersen (2013); Battini et al. (2014); Knapp et al. (2014); Llonch et al. (2017); Mohankumar Sajeev et al. (2018).

^†^Inconsistent/variable results.

^‡^Uncertainty due to limited research or lack of data.

### Enteric fermentation

Decreasing methane emissions from ruminants is one pressing challenge facing the ruminant production sector. Strategies for reducing this source of emissions focus on improving the efficiency of rumen fermentation and increasing animal productivity. A large number of mitigation options have been proposed (e.g., diet manipulation, vaccines, chemical additives, animal genetic selection, etc.) with different efficiencies in reducing enteric methane as shown in Table 1.

Forage quality and digestibility affect enteric methane production. Lignin content increases during plant growth, consequently reducing plant digestibility. Therefore, harvesting forage (especially grass) for ensiling at an earlier stage of maturity increases its soluble carbohydrate content and reduces lignification. According to Knapp et al. (2014) practices aimed to increase forage quality have shown a potential enteric methane reduction of about 5% per unit of fat protein corrected milk.

Physical processing of forages, such as chopping, grinding, and steam treatment, also improves forage digestibility and mitigates enteric methane production in ruminants (Hristov et al., 2013). However, the reduction potential of this practice was reported to be less than 2% per unit of fat protein corrected milk (Knapp et al., 2014).

Improving diet digestibility by increasing concentrate feeding is another effective mitigation strategy, reducing by 15% methane emissions per unit of fat protein corrected milk (Knapp et al., 2014). However, the ratio of forage to concentrate has to be carefully taken into account when applying this strategy. Indeed, although a marked reduction of enteric methane can be expected with rates of concentrate inclusion between 35% and 40% (Gerber et al., 2013). A greater proportion of dietary fermentable carbohydrates could increase the risk of metabolic diseases (e.g., rumen acidosis).

Addition of fats or fatty acids to the diets of ruminants can decrease enteric methane emissions by both decreasing the proportion of energy supplied from fermentable carbohydrates and changes in the microbial population of the rumen (Llonch et al., 2017). Although some byproducts (e.g., cottonseed, brewer’s grains, cold-pressed canola meal, etc.) are effective in reducing enteric fermentation (Moate et al., 2011), the mitigation potential of high oil byproducts has not been well-established and in some cases methane production may increase due to increased fiber intake (Hristov et al., 2013). The inclusion of lipids higher than 10% can lead to impairment of ruminal function due to changes to the microbial population which in turn decreases the ability to digest fiber. Lipid diet supplementation between 5% and 8% of the dry matter intake is an effective mitigation strategy (Grainger and Beauchemin, 2011) with a potential enteric methane reduction of about 15% per unit of fat protein corrected milk (Knapp et al., 2014).

Feed additives (electron receptors, ionophoric antibiotics, chemical inhibitors, etc.) have also been tested for their ability to decrease methane emissions (Beauchemin et al., 2009). However, the unknown toxicity and the health risks associated with the use of some of these compounds may severely constrain widespread adoption (Herrero et al., 2016).

### Manure storage

Increased animal density together with continuous inflow of nutrients from imported feeds is likely to increase volumes of manure to be managed. Stored manure accounts for a relatively small amount of direct agricultural greenhouse gases (Figure 2), and it is technically possible to mitigate a very high percentage of these emissions (Hristov et al., 2013). In the following section, some of the most effective mitigation strategies are discussed.

As methane production increases with the temperature of stored manure, a reduction of storage temperature has been reported to drop these emissions by 30–50% (Borhan et al., 2012). However, the net greenhouse gas mitigation resulting from this strategy can vary widely, and it is strictly related to the energy used and the cooling system adopted.

Frequent removal of manure to an outside storage facility is an effective practice that can be accomplished using grooved floors combined with regular scraping of manure, especially for pigs and some cattle production systems. Indeed, if the channels underneath the stable are emptied regularly, and the manure/slurry are transported to an outside storage facility, this practice has the potential to reduce methane and nitrous oxide emissions by 55% and 41%, respectively (Mohankumar Sajeev et al., 2018). On poultry farms the litter/manure is usually removed at the end of the crop; however, advanced layer housing using belt scrapers can efficiently remove litter/manure continuously and decrease greenhouse gas emissions (Fournel et al., 2012).

Solid-liquid separation is a processing technology that partially separates the solids from liquid manure using gravity or mechanical systems such as centrifuges or filter presses. As shown in Table 1, the greenhouse gas mitigation potential of this technique has been reported to be higher than 30% compared with untreated manure (Montes et al., 2013). The organic component with a larger particle size follows the solid stream during the separation process, and it is then stored in stockpiles. The aerated condition of the storage can then limit the potential for methane to be emitted; however, ammonia loss through composting and generating high temperatures can be accelerated. Also, the remaining liquid fraction is still a potential source of indirect nitrous oxide emissions. Indeed, once the fibrous and large pieces of organic material are subtracted, it will not form a crust during storage, leading to increased volatilization of ammonia by increasing the mass transfer coefficient at the surface. Although greenhouse gas mitigation of the solid-liquid separation process can be partially counterbalanced by ammonia emissions, it is important to note that there are many management practices that can overcome these issues, such as covering slurry storage and the use of injection for land application (Holly et al., 2017).

Anaerobic digestion is a biological degradation process, which in the absence of oxygen, produces digestate and biogas (mainly methane and carbon dioxide) from manure. Biogas collected from the system is often used to generate electricity, to fuel boilers or furnaces, or to provide combined heat and power. Taking into account the greenhouse gas emissions arising from the use of the digestate as fertilizer, and the credit for the renewable energy produced, anaerobic digestion has been reported to yield more than 30% reduction in greenhouse gas emissions when compared with traditional manure handling systems (Battini et al., 2014). However, further attention to the management of the digestate leaving the anaerobic digestion is needed. Indeed, mineralization of the organic nitrogen occurring during biological degradation increases the inorganic nitrogen content and pH of the effluent, which in turn may increase ammonia volatilization (Petersen and Sommer, 2011). Combining anaerobic digestion and solid-liquid separation could reduce the amount of ammonia lost following digestion (Holly et al., 2017).

Diet severely affects excretion of nitrogen in most farm animals, therefore grouping livestock on the basis of their feed requirements can help in reducing this source of nitrous oxide in the excreta. Although a low-protein diet could effectively mitigate nitrous oxide emissions from cattle manure storage (Table 1), some attention must be given to manipulating dietary nitrogen (Montes et al., 2013). For example, decreasing protein could lead to an increase of fermentable carbohydrates, which in turn will likely increase methane production.

The diet for all animal species should be balanced for amino acids to avoid a depression in feed intake and a decrease in animal productivity. Manufactured amino acids are routinely used to balance the diet of monogastrics (pigs and poultry), but the environmental impact associated with the manufacturing of these supplements must be considered when including amino acids as a greenhouse gas mitigation strategy. In ruminants, supplementation of free amino acids results in fast degradation in the rumen, without a significant increase in animal productivity. On the contrary, rumen-protected amino acids resist chemical alterations in the rumen and can reach the intestine where they are absorbed, improving milk yield in dairy cows. Overall, feeding protein close to the animal’s requirement is recommended as an effective mitigation strategy to reduce ammonia and nitrous oxide emissions from manure (Montes et al., 2013).

### Feed production

The timing, quantity, and method of fertilizer applications are important factors influencing soil nitrous oxide emissions. The nitrogen fertilizer applied is susceptible to loss by leaching and denitrification before crop uptake. Therefore, ensuring that appropriate amounts of nitrogen get to the growing crop and avoiding application in wet seasons or before major rainfall events, are valuable practices which could help in optimizing biomass production and reduce soil greenhouse gas emissions.

As lower methane emissions occur after manure land application, decreasing storage time can effectively help in reducing greenhouse gas emissions (Table 1). However, the resulting frequent soil applications can have a variable effect on nitrous oxide emissions from field and carbon dioxide emissions from fuel combustion. Avoiding application during prolonged periods with wet soil and periods of low plant nitrogen uptake could help in increasing the effectiveness of this practice (Hristov et al., 2013).

Adequate storage facilities can provide greater flexibility in choosing when to apply manure to fields, while the use of on-farm manure analysis could help the farmer develop a nutrient management plan and minimize environmental impacts (Newell Price et al., 2011).

The use of nitrification inhibitors has the potential to reduce nitrogen leaching by inhibiting the conversion of ammonia to nitrate. However, this beneficial effect is weakened by a reported increase in indirect nitrous oxide emission that can result from increased ammonia volatilization (Lam et al., 2016). This highlights the importance of considering both gases when evaluating the use of nitrification inhibitors as an option to mitigate climate change. Overall, nitrification inhibitors have been demonstrated as an effective practice to reduce nitrous oxide emissions (Table 1).

Intensive rotational grazing systems are being promoted as a good way to increase forage production and reduce nitrous oxide emissions (Table 1). These systems are characterized by multiple smaller fields called paddocks for the rotation of livestock. By subdividing pastures and rotating animals, farmers can manage stocking densities and grazing duration and thereby manage nitrogen excreta distribution and vegetation regrowth. A more uniform distribution of urine throughout the paddock would reduce the effective nitrogen application rate, which could translate into a reduction in nitrous oxide emissions (Eckard et al., 2010). Keeping animals off the paddocks during wet weather will reduce sward damage and soil compaction. In addition, avoiding excreta deposition at these times will reduce nitrous oxide emissions and nitrogen leaching (Luo et al., 2010).

### Animal management

There is a direct link between greenhouse gas emission intensities and animal efficiency. The more productive the animal is, the lower the environmental impact will be (on a per unit of product basis). Both management quality and expression of full genetic potential are necessary to increase production efficiency.

Breeding for more productive animals can lead to a reduction of the nutrient requirements needed to reach the same level of production. This is a valuable greenhouse gas mitigation strategy (Table 1). A more efficient animal will retain more dietary nitrogen protein and there will less nitrogen in feces and urine (Gerber et al., 2013). Genetic improvement of daily gain and feed conversion that has been achieved in broilers over the last 20 years has reduced substantially the emissions per unit of weight (Williams and Speller, 2016). Nevertheless, strategies that aim to change animal phenotypes to enhance productivity or efficiency may harm animal health and welfare unless these effects are measured and controlled (Llonch et al., 2017). Animals of a particular genotype selected for increased production will only be able to realize this potential on a high input system in which resources are adequately supplied. In other words, new breeds and crosses can lead to substantial greenhouse gas reduction, but they need to fit within production systems and climates that may be characterized by limited resources and other constraints.

Poor fertility means that more breeding animals are required in the herd to meet production targets, and more replacements are required to maintain the herd size, which in turn increases greenhouse gas emissions. Improved fertility in dairy cattle could lead to a reduction in methane emissions by 10–24% and reduced nitrous oxide by 9–17% (Table 1). Nevertheless, increasing reproductive pressure may increase the metabolic demands associated with pregnancy and lactation that could negatively affect animal health and increase the risk of metabolic diseases, reduce immune function and in turn reduce fertility (Llonch et al., 2017).

Poorer livestock health and welfare are associated with behavioral and metabolic changes, which can effect greenhouse gas emissions in several ways. Animals fighting an infection will need more energy for maintenance. A recent study in the United Kingdom investigated cost-effective ways to reduce greenhouse gas emissions by improving cattle health. These studies found that cattle diseases can increase greenhouse gas emissions up to 24% per unit of milk produced and up to 113% per unit of beef carcass (Williams et al., 2015). A disease that temporarily reduces feed intake or the ability to digest feed, leads to a decline in growth rate, which will result in more time and energy needed to reach the same end point.

## Conclusion

Agriculture in general, and livestock production, in particular, contributes to global warming through emissions of methane and nitrous oxide. To meet future needs of an expanding population, animal productivity will need to increase and greenhouse gas emission intensity per unit of product will need to decrease. One of the principal ways to achieve this environmental standard is to adopt effective mitigation strategies. To increase the effectiveness of these strategies, complex interactions among the components of livestock production systems must be taken into account to avoid environmental trade-offs. Unfortunately, there is not a standard procedure to follow. Mitigation practices should not be evaluated individually, but as a component of the entire livestock production system. The majority of these strategies aim to increase productivity (unit of product per animal), which in most cases cannot be achieved without good standards of animal health and welfare. Optimizing animal productivity has a powerful mitigating effect in both developed and developing countries; however, the size of the effect will also depend on factors such as the genetic potential of the animal and adoption of management technologies.

## About the Authors

**Giampiero Grossi** is a PhD student in the Department of Agriculture and Forestry Science (DAFNE) at Tuscia University, Italy. His research is focused on the quantification of greenhouse gases arising from a typical agro-silvo-pastoral system of the Mediterranean area. Giampiero is currently applying life cycle assessment methodology to a case study in Castelporziano, Rome. His background encompasses agri-food environmental certifications, livestock management, and farming practices.

**Pietro Goglio** is a lecturer in life-cycle assessment and systems modeling at Cranfield University. He has a strong environmental background and has conducted research in the life-cycle analysis of agricultural and bioenergy systems. Currently, Dr Goglio is focusing his research on developing approaches to combine science with life cycle assessment approaches for greenhouse gas removal from the atmosphere and for greenhouse gas accounting for agricultural systems and food systems. These research developments aim to better capture the characteristics of the systems by considering the economic, social, and political factors affecting their performance and implementation.

**Andrea Vitali** is a lecturer in Sustainable Livestock Production in the master degree of Food Science and Technology at University of Teramo. His research focused on the bidirectional relationships between animals and the environment. He has studied the effects of heat stress on livestock (production, reproduction, and health) and the contribution of animals to global warming. He has expertise in the application of systems based life-cycle assessment to livestock production. He was involved in developing the Italian plan for adaptation to climate change related to agriculture and food production.

**Adrian Williams** has spent many years working in agri-environmental science. He is a leading expert in the application of systems based life-cycle assessment to agricultural and food production. He has studied the production of all major crop and livestock species in the United Kingdom and abroad (e.g., beef in Brazil). He has applied life-cycle assessment to the greenhouse gas benefits of improved cattle health as well as enhanced welfare in pig and poultry housing. He is responsible for developing the beef sector model in the recently enhanced agricultural greenhouse gas inventory in the United Kingdom.
